# Role of Smoking in the Pathogenesis of Multiple Sclerosis: A Review Article

**DOI:** 10.7759/cureus.9564

**Published:** 2020-08-05

**Authors:** Katukuri Nishanth, Ezza Tariq, Farirai P Nzvere, Mohammed Miqdad, Ivan Cancarevic

**Affiliations:** 1 Internal Medicine, California Institute of Behavioral Neurosciences & Psychology, Fairfield, USA; 2 Internal Medicine, Nishtar Medical College, Multan, PAK

**Keywords:** cigarette smoking, multiple sclerosis, epigenetics, epidemiology, risk factors

## Abstract

Multiple sclerosis (MS) is a complex and unpredictable neurological condition. It is the most commonly seen autoimmune disorder. The incidence of disease and its prevalence are growing worldwide. Early identification of the disease and accurate diagnosis is important to prevent further complications and disability. The etiology remains unclear, and it is believed that complex gene-environment interactions play an essential role. Genetic predisposition only describes a portion of the disease risk, whereas lifestyle and environmental factors are significant contributors. Smoking was identified as an important risk factor for MS. The main objectives of this review were to examine the underlying mechanisms of immune dysregulation in the development of MS, explore the association between smoking and MS, and identify other genetic and environmental factors that alter the risk of developing the disease. We searched PubMed for articles relevant to the study topic published between 2000 and 2020 using the search terms “multiple sclerosis,” “cigarette smoking,” “risk factors,” and, “epigenetics.” Studies reveal a marked association between smoking and the risk of MS. Unlike genetic risk factors, many lifestyles and environmental factors can be adjusted, with potential for prevention, particularly for people at the highest risk, such as families of individuals with MS.

## Introduction and background

Multiple sclerosis (MS) is the most common autoimmune inflammatory demyelinating disorder of the brain and spinal cord [1]. It is a widespread debilitating and disabling neurological disease commonly seen in young adults worldwide [2]. Most often, people with MS are diagnosed between 20 and 40 years of age. MS patients commonly experience urinary symptoms, pain, sexual problems, tiredness, visual disturbances, muscle stiffness, spasticity, changes in cognitive function, and depression. The course of the disease is very unpredictable in such a way that a small number of patients with MS have a mild course of infection with little to no disability; on the other hand, some people develop a steadily worsening disease that leads to increased disability over time. Environmental factors like smoking, low vitamin D, Epstein-Barr virus (EBV) infection, childhood obesity, and lack of physical activity are some of the risk factors for MS [1]. Four basic disease courses have been identified in MS: clinically isolated syndrome (CIS), primary progressive MS (PPMS), relapsing-remitting MS (RRMS), and secondary progressive MS (SPMS) [3]. 

CIS refers to the first acute episode suggestive of inflammation and demyelination in the central nervous system (CNS) [3]. CIS may be the first presentation of the illness. The risk of developing MS following CIS is very high. When CIS is associated with lesions on brain MRI similar to the ones seen in MS, there is a higher chance for a second neurological event and diagnosis of clinically definite MS (CDMS). Individuals who experience CIS alone with no evidence of MS-like lesions on brain MRI are at relatively low risk of developing MS. RRMS affects about 85% of patients and is defined by relapses lasting from days to weeks, followed by complete or partial remission over months or years [1]. PPMS affects approximately 10% of the patients. PPMS is marked by a progressive accumulation of disabilities following the disease's initial relapse; symptoms tend to deteriorate and become more resistant to drug therapies [1-3]. SPMS follows an initial relapsing-remitting path [1]. Nearly 75% of patients with RRMS will transition to SPMS within 15 years of initial diagnosis [4]. Besides progressive worsening of neurological functions, treatment with disease-modifying agents may delay the progression of RRMS to SPMS [1,4].

Currently, there is no cure, and researchers are still trying to find the cause of the disease [2]. Both genetic and environmental factors play an important role in developing MS [5]. New medicines and therapies developed in the last few years show promising results in slowing down the progression of the disease, disability, and complications [2]. Researchers see promising results with drugs and therapies that are designed to protect myelin cells against damage or that could help them recover after injury. On 25 March 2020, US Food and Drug Administration approved drug Zeposia (ozanimod) oral capsules to treat adults with relapsing forms of MS [6]. NINDS (National Institute of Neurological Disorders and Stroke) sponsored a clinical trial to know whether coupling two therapies, glatiramer acetate, and beta-interferon are beneficial for preventing relapses [2]. 

The number of MS cases has significantly increased since 1990, and recent studies estimate that more than 2.5 million people have this condition globally [2]. Nearly 250,000-300,000 people diagnosed with MS in the United States. Environmental factors, like smoking, are risk factors for the development, severity, progression, disability, and early death in MS [5,7-9]. In this review, we are going to discuss MS potential etiological factors, epidemiology, and pathology.

## Review

Damaging effects of cigarette smoke on the immune system in MS

The effects of cigarette smoke on the immune system are not well known [10]. Tobacco contains high amounts of free radicals. It has been reported that free radical-induced oxidative stress causes mutations in genetic material and is involved in several neurodegenerative disorders, such as Parkinson's disease and MS. Tobacco increases the risk of autoimmune diseases, such as systemic lupus erythematosus and rheumatoid arthritis, and is associated with other autoimmune disorders like MS [11,12]. Cigarette smoke acts on the cellular level of the immune system, resulting in the development of proinflammatory cytokines [11-13]. Smokers have high levels of proinflammatory cytokines (e.g., IL-6), higher levels of C-reactive protein, fibrinogen, and other inflammatory markers [11]. Sustained, elevated levels of proinflammatory cytokines could contribute to persistent autoimmunity. In general, people with MS have higher levels of NO, due to the presence of an inducible form of nitric oxide synthase (iNOS) in cells, such as macrophages and astrocytes [14]. The elevated levels of NO can cause mitochondrial damage, oligodendrocyte necrosis, and axonal degeneration, ultimately leading to impairment in axonal conduction [14,15]. Exposure to harmful gases like carbon monoxide (CO) affects tissue oxygenation and can lead to demyelination [7]. Carbon monoxide (CO) neurotoxicity occurs through a number of mechanisms including lipid peroxidation, inflammatory, myelin degeneration, immune-mediated reactions, and finally neuronal apoptosis and necrosis. Smoking decreases the numbers of peripheral B cells and natural killer (NK) cells, decreases the production of cytokines by antigen-presenting cells, and reduces the blood levels of serum immunoglobulins [11]. Nitric oxide (NO) and carbon monoxide (CO), in cigarette smoke, enhance the inflammatory response and attenuate some of the immune defenses resulting in increased susceptibility to infections [7,13]. These changes in the immune system explain a few possible mechanisms for increased susceptibility to viral respiratory tract infections in MS patients [7,11,13]. Respiratory infections are an essential trigger for relapse in MS [11]. Sibley et al. found the risk of relapse was increased almost threefold in two weeks prior and three weeks post-respiratory viral infection [16]. Tobacco smoking lessens the activation of CD4^+^ T cells, modifies antigen-mediated T-cell signaling, and causes inactivation of CD8^+^ T cells in response to infection [17]. 

Additionally, smoking increases the expression of Fas (CD95), a cell surface molecule on CD4^+^ T cells and B cells, and makes these cells more sensitive to apoptosis [18]. Increased accumulation of apoptotic material may be significant if clearance mechanisms are weakened in individuals with autoimmunity. Cigarette smoke induces irritation in the lung and may trigger the development of autoaggressive T cells in the lungs, which are cross-reactive with CNS antigens and initiate a CNS-directed autoaggressive immunity resulting in MS [19]. In the tobacco, a polyphenol-rich glycoprotein stimulates the proliferation of T cells and differentiation of B cells, creating a proinflammatory environment [7]. Proinflammatory cells like G-protein-coupled receptor 15 (GPR15) T cells are increased in smokers and associated with MS [20]. Acrolein and hydrogen cyanide in cigarette smoke can induce immunosuppression and neurodegeneration [7,15]. 

To sum up, smoking is a major environmental risk factor for the incidence of MS, and it plays a vital role in immunological mechanisms. Data from multiple studies show that tissue injury in MS is due to an abnormal immune response to one or more myelin antigens that develops in genetically susceptible individuals after exposure to the risk factors like cigarette smoke. Therefore, stopping smoking could alleviate atypical immune responses and tissue damage.

Epigenetics of MS

Epigenetics play a crucial role in the pathogenesis of MS [21]. Current research implies that the interplay between genetic and environmental factors is necessary for disease manifestation [22]. The smoking status may strongly influence the risk of developing MS associated with human leukocyte antigen (HLA) genotypes [23]. HLA-DRB1*15:01 and HLA-A*02 are considerable risk and protective variants for MS, respectively [15,19]. Smokers carrying HLA-DRB1*15 and lacking HLA-A*02 had a 14-fold increased risk compared with non-smokers [15,19,23]. The inheritance pattern of MS is still unknown, although the condition appears to pass down through generations in families [24]. Incidence and prevalence of MS are higher among the siblings or children of a person with the disease than for the general population. There is a higher clinical concordance rate (25%-30%) among monozygotic or identical twins than dizygotic or fraternal twins (3%-7%) [25]. HLA-DRB1*15:01, genetic risk score (GRS), and smoking were associated with earlier onset of disease [26]. A significant interaction between NAT1 variant rs7388368, HLA-DRB1*15:01, and HLA- A*02 was observed among smokers [23,27]. In contrast, such an association was absent among non-smokers [23]. Genome-wide association studies (GWAS), a new study design introduced in the mid-2000s, revolutionized the genetics of MS [22]. To date, the most extensive genetic study has identified 233 genome-wide loci associated with MS susceptibility [25]. Out of 233 loci, 200 of these loci explain ∼20% of the genetics [25]. Increased or decreased acetylation, methylation, and citrullination of genes controlling the expression of inflammation and myelination factors appear to be mainly involved [21]. HLA-DRB1*15:01 allele is the significant risk factor for MS and regulated by epigenetic mechanisms, such as DNA methylation and histone deacetylation. Some pieces of evidence support that blood DNA methylation is affected by cigarette smoke [28]. Smoking causes DNA methylation in MS patients in an exposure-dependent manner [23,28]. Hypomethylation is more pronounced in current smokers or those who ceased smoking less than five years ago [28]. In comparison, methylation levels in MS patients who stopped smoking more than five years ago are comparable to those in non-smokers. 

Genetic predispositions, along with environmental factors, play an essential role in the pathogenesis of the disease. The wide heterogeneity in etiology, together with the varying responses to treatment, signifies further hurdles to understand and treat the underlying condition. Over the last decade, we have seen real progress in defining the genetic basis of MS. Identifying and characterizing MS susceptibility genes and their correlation with disease phenotypes is likely to identify the underlying etiology of the disease, improve risk assessment, and influence therapeutics.

Anatomical and cognitive deficits in MS patients

Smoking affects the cognitive functions of MS patients [29,30]. The prevalence of cognitive deficits in patients with MS ranges from 40% to 70% [29]. Memory is most often affected, and the number of patients that experience memory and learning problems is between 40% and 60% [31]. Cognitive deficits may occur depending on the size and location of the focal area of demyelination in the CNS [30]. Studies have reported that male MS patients have more significant cognitive impairments than females, but in recent research, it was found that sex is not associated with cognitive impairment [32,33]. A few studies were conducted to determine if nicotine or the tar in tobacco smoke was responsible for the adverse effects [30]. These studies concluded that nicotine had positive effects on cognition. Exposure to high concentrations of tar in cigarette smoke may be causing cognitive problems in MS patients. Heavy smokers had increased cognitive impairment when compared to non-smokers. Brain MRI obtained from smokers with MS showed a higher number of plaques, reduced gray matter fraction, lower brain parenchymal fraction, and increased cerebrospinal fluid fraction compared to those who have never smoked or stopped smoking [34,35]. In contrast, no effect was observed on white matter fraction [34]. A decrease in brain volume, especially gray matter, is a strong predictor of long-term physical disability and cognitive issues.

Cigarette smoking has both positive and negative cognitive effects, and due to the increasing incidence of cognitive impairment in MS patients, it is important to explain the cognitive effects of smoking in these patients. Brain volume measures will likely be used as predictors of treatment response and disease progression. They will also have a significant impact on treatment decisions, mainly to the development of new MS drugs that could prevent brain atrophy. Understanding decreases in brain matter can become necessary for MS management. Therefore, patients should be advised to avoid smoking to reduce exposure to substances such as tar in cigarette smoke. 

Smoking and disease progression in MS

Smoking adversely influences the course of the disease [7,36]. According to the studies mentioned in Table 1, smokers have an elevated risk of developing MS compared to non-smokers [37-40]. Research suggests that both the duration (years) and intensity of tobacco smoking also affect the risk of developing the disease [40,41]. Current cigarette smokers had more severe disease at baseline than never-smokers in terms of Expanded Disability Status Scale score (EDSS) [37]. Exposure to pollutants present in tobacco-related products contributes to the development of proangiogenic and pathogenic CCR6^+^Th17 cells [12]. These cells worsen chronic inflammatory disorders, such as MS and rheumatoid arthritis [12]. Di Pauli et al. study reported that nicotine users diagnosed with the CIS develop a clinically definite course of the disease (CDMS) in a shorter period than non-smokers [42]. A large prospective cohort study conducted by Healy et al. suggests that patients who quit smoking may not only decrease the risk of developing smoking-related complications but also delay the progression of MS [37]. Patients with this condition are sensitive to the central effects of smoking, resulting in an acute worsening of symptoms like blurred vision, motor weakness, and paresthesias [36]. Cigarette users diagnosed with this autoimmune disorder experience more severe symptoms, an increased number of relapses, and more significant disability compared to non-smokers [43]. Data from multiple studies suggest that cigarette smoke has an influence on disease progression and accelerates the conversion from a relapsing-remitting to a progressive course (SPMS) of MS [37,43,44]. Ramanujam et al. found that the risk for transition to a secondary progressive form of the disease was increased by 4.7% per year for each additional year of smoking after the diagnosis of RRMS [45]. Pittas et al. stated patients who had ever smoked were twice likely to develop the progressive form of the condition [44]. Maghzi et al. found that an ex-smoking habit could increase the risk of MS by 8.83-fold [46]. Sundström and Nyström reported that people who started smoking early (<15 years of age) were more likely to develop progressive forms of the disease [47]. In a three-year prospective cohort study conducted in southern Tasmania, it was determined that using cigars was positively associated with clinical disability progression [44]. 

**Table 1:** Key studies on the association between smoking and MS.

Study	Study Design	Main Outcome
Study 1 [38]	A prospective cohort study examined 3,052 cases against 457,619 controls.	Reported a relative risk (RR) of 1.48 and concluded that smoking might increase the susceptibility to multiple sclerosis (MS).
Study 2 [23]	A case-control study with 843 cases and 1,209 controls.	Among those with genetic risk factors, smoking increased the risk by a factor of 2.8 in comparison with a factor of 1.4 among those without the genetic risk factors.
Study 3 [46]	This case-control study compared 516 cases with 1,090 controls.	Past smokers and current smokers had a significant risk of developing MS.
Study 4 [39]	A low-quality case-control study in which 81 patients with MS were compared with 81 paired controls.	This study stated that ever smoking could increase the risk of MS by 7.6-fold.
Study 5 [40]	Two Swedish population-based case-control studies consisted of 7,883 cases and 9,264 controls.	This research saw a clear dose-response association between cumulative dose of smoking and MS risk.
Study 6 [37]	This is a cross-sectional survey and longitudinal follow-up of 1,465 patients with clinically definite MS.	MS in smokers progressed from relapsing-remitting (RRMS) to secondary progressive disease (SPMS) faster than in never-smokers.
Study 7 [43]	Retrospective research consisted of 179 cases with a mean follow-up period of 5.3 years.	These results suggest that smoking may be a risk factor for transforming a relapsing-remitting (RRMS) course into a secondary progressive course (SPMS).
Study 8 [47]	Smoking habits in 122 cases were assessed with a median of six years of disease duration.	Compared with never-smokers, ever-smokers were more likely to have a progressive illness.
Study 9 [41]	Smoking status is assessed in a case-control study consisting of 210 cases with clinically proven or laboratory-confirmed MS.	A dose-response relationship between the risk of MS and both duration (years) of smoking and the number of cigarettes smoked daily was observed.
Study 10 [27]	In Sweden, two population-based case-control datasets were used to perform a three-stage gene analysis for NAT1, NAT2, and GSTP1 variants.	They found out NAT1 as a genetic effect modifier of tobacco smoke exposure in MS susceptibility.

Studies described in Table 1 provide clinicians with valuable data by showing that individuals affected by MS who are actively smoking are more likely to experience a more severe clinical course with significant deficits in all domains. Stopping tobacco use could delay the onset of symptoms, disability development (use of a walker, or assistive device), and reduce the number of relapses and complications. 

## Conclusions

MS is a complex neurological disease in which the interplay between genetic and environmental factors triggers a cascade of events, including activation of the immune system, CNS demyelination, and neuronal degeneration with variable degrees of repair. In this article, we have analyzed the association between smoking and MS in different population groups.​ Smoking should be considered as a modifiable risk factor for MS, and it may account for some of the excess mortality among patients with MS. The development of therapeutics incorporating neuroprotection and remyelination to treat and eventually prevent the disabling and progressive forms of the condition is a challenge. Early diagnosis and treatment could help prevent the complications and disability associated with MS. Health promotions aimed at smoking cessation must be encouraged. More research needs to be done to improve our understanding of the possible links between tobacco and MS, to investigate the harmful effects of tobacco on mentation, to determine the impact of exposure to second-hand smoke, and to assess whether smoking affects the patient's response to immunomodulatory therapy.
